# Assessing the Ecosystem Health of Coastal Wetland Vegetation (*Suaeda salsa*) Using the Pressure State Response Model, a Case of the Liao River Estuary in China

**DOI:** 10.3390/ijerph19010546

**Published:** 2022-01-04

**Authors:** Ziming Song, Yingyue Sun, Peng Chen, Mingming Jia

**Affiliations:** 1 College of Tourism and Geography Science, Jilin Normal University, Siping 136000, China; pz2@jlnu.edu.cn (Z.S.); chenpeng@jlnu.edu.cn (P.C.); 2 Key Laboratory of Wetland Ecology and Environment, Northeast Institute of Geography and Agroecology, Chinese Academy of Sciences (NEIGAE), Changchun 130102, China; jiamingming@iga.ac.cn

**Keywords:** coastal wetland, *Suaeda salsa*, pressure-state-response framework, ecological restoration assessment

## Abstract

*Suaeda salsa* (*S. salsa*) is an important ecological barrier and tourism resource in coastal wetland resources, and assessing changes in its health is beneficial for protecting the ecological health of wetlands and increasing finances. The aim was to explore improvements in the degradation of *S. salsa* communities in the Liao River Estuary National Nature Reserve since a wetland restoration project was carried out in Panjin, Liaoning Province, China, in 2015. In this study, landscape changes in the reserve were assessed based on Sentinel-2 images classification results from 2016 to 2019. A pressure-state-response framework was constructed to assess the annual degradation of *S. salsa* communities within the wetlands. The assessment results show that the area of *S. salsa* communities and water bodies decreased annually from 2016 to 2019, and the increased degradation indicators indicate a state of continued degradation. The area of types such as aquaculture ponds and *Phragmites australis* communities did not change much, while the estuarine mudflats increased year by year. The causes of *S. salsa* community degradation include anthropogenic impacts from abandoned aquaculture ponds and sluice control systems but also natural impacts from changes in the tidal amplitude and soil properties of the mudflats. The results also indicate that the living conditions of *S. salsa* in the Liao River estuary wetlands are poor and that anthropogenic disturbance is necessary to restore the original vegetation abundance.

## 1. Introduction

The *Suaeda salsa* (*S. salsa*) community is a common vegetation in coastal wetland ecosystems, usually growing in the shallows near the coast, and is highly salt tolerant. *S. salsa* is widely distributed in coastal and north-western China, Central Asia and Europe, and it has a role in improving the physical and chemical properties of soils, providing habitat for animals, coping with climate change and maintaining the ecological functions of wetlands [1,2,3,4,5]. The Liao River Estuary National Nature Reserve in China is also home to a community of *S. salsa*, which grows in April and May each year and turns from green to red in August and September, creating the famous ‘red beach’ landscape, which has become an important ecotourism resource for increasing revenue [5].

However, from 1988 to 2009, the entire wetland has been in a state of degradation due to natural and human factors (e.g., aquaculture ponds and drought), especially the ‘red beach’ landscape consisting of the *S. salsa* community, which not only constrains the development of the local tourism industry but also threatens the wetland’s ecological health [6,7]. Degradation also exists in other coastal wetlands in China, such as the Yellow Sea [8]. In recent years, the shrinking of the “red beach” has received widespread attention from the government, and, in 2015, a wetland ecological restoration project was launched to restore the beach to its original state. However, there have been few reports on whether the degradation of the *S. salsa* community has improved after the implementation of the project. Evaluating the degradation of the *S. salsa* community objectively is of great importance for the scientific protection and management of the Liao River estuary wetland.

Researchers often develop models or frameworks to assess the ecosystem health of landscapes such as rivers, lakes and wetlands to provide a scientific reference. At present, the commonly used ecosystem evaluation models or frameworks include the Analytic Hierarchy Process (AHP) model [9,10,11,12], the comprehensive evaluation model based on entropy weight [13,14,15] and the pressure state response (PSR) frameworks [16,17,18]. The AHP method and entropy weighting method are mainly used to determine indicator weights [bai18]. Many studies have used the AHP method to determine the weights of individual indicators before constructing PSR models to assess ecosystem health, including many wetland types, such as plains [19], river basins [16], bays [12], mangroves [17] and so on. The health of wetlands can be easily assessed by PSR models, and the causes of wetland degradation can be identified as agricultural expansion or otherwise [20]. Thus, the pressure state response evaluation model has the advantages of strong internal logical relationships and clear causal relationships, making it suitable for the study of *S. salsa* community degradation in the Liao River estuary. In summary, previous studies found that PSR models can successfully assess wetland ecosystem health issues, but their application to coastal wetlands has been less common, especially for single vegetation species.

Accurate spatial and temporal distribution information about the *S. salsa* community is required for degradation assessment. However, the environmental conditions of coastal wetlands are complex, and it is difficult to carry out a traditional field investigation. Remote sensing methods have the advantages of saving manpower, material resources and financial resources and are a more effective method for coastal area monitoring [21,22,23,24]. Sentinel-2 A and B satellite data, which were put into use in 2015 and 2017, have the advantages of high spatial resolution (10 m), a short revisit period (3 to 5 days), rich waveband, a large image coverage area and free access, and have broad application potential in wetland change research [25,26,27]. There have been many successful cases of using Sentinel 2 satellite data to monitor or assess the condition of wetlands, e.g., Mahdianpari et al. [28] used Sentinel-1 and Sentinel-2 data on the Google Earth Engine cloud computing platform to produce the first wetland inventory map of Newfoundland at a spatial resolution of 10 m. Chatzaintoniou et al. [29] based on co-orbital Sentinel 1 and 2 using machine learning algorithms to map the land use/land change of wetlands in the Mediterranean Sea. Compared to MODIS and Landsat imagery data, the Sentinel series has richer spectral reflectance information and higher spatial resolution for more suitable and clear monitoring of surface changes in small-scale areas. Additionally, an application in classifying wetland land cover found that the machine learning algorithm random forest slightly outperformed support vector machines in concert with Sentinel 1 and 2 [30]. Zhang et al. [31] successfully classified wetland vegetation in the Yellow River estuary using a random forest classification model based on spatio-temporal spectral multidimensional features using Sentinel 2 data. Recently, Heimhuber et al. [32] developed a new open-source Python toolkit for historical and near real-time monitoring of inlet wetlands using Landsat and Sentinel-2 images. Overall, Sentinel-2 is a high-quality source of satellite data and is increasingly valued in the assessment and monitoring of wetlands.

The objective of this study was to assess the health of *S. salsa* communities in the Liao River Estuary National Nature Reserve using the PSR model and Sentinel-2 satellite imagery. Firstly, the surface landscape evolution of the Liao River estuary wetlands is explored based on Sentinel-2 satellite imagery and an object-oriented random forest machine learning classification algorithm. Then, the PSR model was used to assess the health of the *S. salsa* community between 2016 and 2019 and to analyze the driving factors. The results provided a data basis and support for the scientific management and protection of wetland ecosystems in the Liao River Estuary National Nature Reserve.

## 2. Materials and Methods

### 2.1. Overview of the Study Area

**Figure 1:**
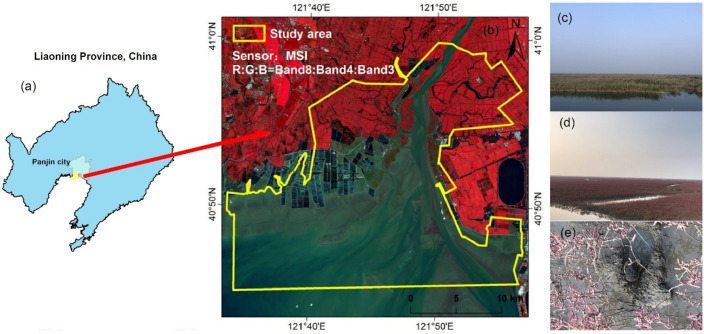
Location of the study area. (**a**) The location of the study area in Liaoning, China. (**b**) Study area boundary, Sentinel 2 satellite image band combinations are able to indicate alkali ponies, with rusty red being *S. salsa* and red indicating vegetation. (**c**–**e**) are field photographs of *P. australis*, *S. salsa* and crab holes within the *S. salsa*, respectively.

The core area of Liao River Estuary National Nature Reserve (121°35′–121°55′ E, 40°45′–41°0′ N) was taken as the study area (Figure 1), located north of Liaodong Bay in the Bohai Sea of China and in the center of the Liao River Delta. The region has a temperate continental monsoon climate, with rainfall concentrated from June to August, an average annual rainfall of 650 mm and an average annual temperature of 8.5 °C. The Liao River wetland, formed by the impact of the Liao River, the Daling River and the Xiaoling River, is an important habitat for more than 30 rare and endangered waterfowls, such as the red crowned crane and the black headed gull. In the study area, *S. salsa*, *Phragmites australis (P. australis)* are the main wetland vegetation types, and other types of low abundance wetlands and artificial vegetation are distributed. Among them, the *S. salsa* community presents bright red foliage continuously from the end of August to the beginning of October each year, which is known as the “red beach”. It is worth noting that *P. australis* has expanded rapidly in the growth zone of *S. salsa* in recent years. In 2019, Panjin City received a total of 35.97 million tourist arrivals, with the main tourist attraction being the Red Beach National Scenic Corridor, located on the east side of the study area. Panjin City launched the implementation of coastal wetland restoration in 2015, covering an area of up to 1533 hectares, including the recovery of the enclosed sea farming ponds, the removal of the enclosed sea dykes, the dredging and dredging of natural tidal ditches, the restoration of tidal channels and the restoration of natural water systems.

### 2.2. Data Sources and Preprocessing

The Sentinel-2 satellite carries a multispectral imager (MSI), which consists of two repeat orbit satellites, A and B. The revisit period is 3 to 5 days. Due to the distinctive red color of *S. salsa* in August and September, four high-quality Sentinel-2 MSI images from 2016 to 2019 were selected as the data sources for evaluating the degradation of *S. salsa* wetlands in the Liao River estuary (Table 1).

These images were downloaded from the ESA data sharing website (https://scihub.copernicus.eu/dhus/#/home (accessed on 5 September 2021)). The product grade was 1C. Using Sen2Cor remote sensing image processing software released by ESA, the 1C data were converted into 2A surface reflectance data. Then, the data of each band of the 2A level were resampled to 10 m using the nearest neighbor interpolation method using snap5.0 software.

The meteorological data used in this paper were obtained from the China Meteorological Data Service Center (http://data.cma.cn/ (accessed on 5 September 2021)). According to the location of the study area, the data from Yingkou station number 54,471 were selected. Precipitation and temperature data were selected from March to October because *S. salsa* is an annual herbaceous vegetation and this time range corresponds to its growing season. Therefore, the average of monthly mean temperature and precipitation data from March to October of each year was used as the average temperature and average precipitation for the growing season of that year.

### 2.3. Remote Sensing Image Classification Method and Classification System

In this study, the object-oriented random forest algorithm was used to classify remote sensing images due to its dependability and stability [27,28,29,30]. The purpose of using this supervised classification algorithm is to obtain the land cover type for each year. The random forest algorithm is a machine learning algorithm based on decision tree combination, and the number of trees in this study is set to 10.

In this study, a land cover classification system of the Liao River estuary was established based on the purpose of the study, the characteristics of land cover in the study area and previous research cases [6]. There were seven types of land cover in Liao River estuary: *S. salsa*, building land (houses and roads), paddy fields, *P. australis*, water (river and other natural open water surfaces), aquaculture ponds and estuarine mudflats (river channels, coastal beaches and sediment deposited at the estuary). The process involves decomposing the image into basic objects to classify optimized feature metrics, after which a classification feature space is applied to the classes, then training samples are created and classification is performed, with the classifier selecting a random forest. The Overall accuracy, Kappa coefficient accuracy is high, but the User accuracy is relatively low (Table 2), so experts were invited to post-process the land cover data such as validation and modification to obtain four high accuracy land cover maps of the Liao River estuary from 2016 to 2019.

### 2.4. Construction of Assessment Index System

According to the ecological environment of *S. salsa* in the Liao River Estuary National Nature Reserve, combined with various methods of wetland degradation and health assessment, this study selected indicators that could truly reflect the degradation characteristics of *S. salsa* with regard to pressure, state and response and formed an evaluation index system (Table 3). The health index system of *S. salsa* in the Liao River estuary wetland included three levels. The pressure indexes included a tidal flat disturbance index, temperature change and precipitation. The disturbance index of tidal flat was expressed by the area converted from river to beach every year. The average temperature and precipitation in spring and summer (March to August) were selected to express the temperature change and rainfall. The Normalised Vegetation Index (NDVI) and the habitat quality index of the *S. salsa* community indicate vegetation vitality. The three landscape indices are indicators that give a good indication of the structural organization of the landscape, and they were calculated by the Fragstats4.2 program. Fragstats 4.2 is a spatial pattern analysis program developed by McGarigal and Marks [33] for quantifying the structure of the landscape and is simple to use. The classification results are entered into the program, the metrics to be calculated are selected and the results are run. Average elasticity indicates the ability of wetland ecosystems to self-regulate and resist various stresses. The response indicators include the ecological protection index and wetland area ratio. In order to objectively evaluate the degradation of *S. salsa* wetlands in the Liao River estuary, data on the area of *S. salsa* are not used as indicators.

### 2.5. Calculation of Index Weight

Determining indicator weights is about determining the contribution of each factor to the evaluation unit score. In this paper, the AHP method is used to assign the weights of each indicator in the indicator system. The AHP method can establish a hierarchical structure of the decision-making process in a complex system and organically combine the qualitative and quantitative factors in the decision-making process. Many studies have been conducted to confirm the generalizability and reliability of AHP in evaluating ecosystem health [34,35,36,37,38,39,40,41]. In this study, the AHP structure was defined as goal layer, criteria layer and indices layer. The evaluation of the *S. salsa* growth condition was set as the goal layer, the pressure and state layer, response index was set as the criteria layer and 13 indices as the indices layer to construct a AHP. Then, the weights of each index were obtained by establishing a judgment matrix, ranking calculations and consistency tests, and the specific process can be referred to in the study of Zhang et al. [42]. The judgment matrix and weights of the AHP method for each indicator of the PSR model are shown in Table 4.

### 2.6. Composite Assessment of the S. salsa Community Health

**Figure 2:**
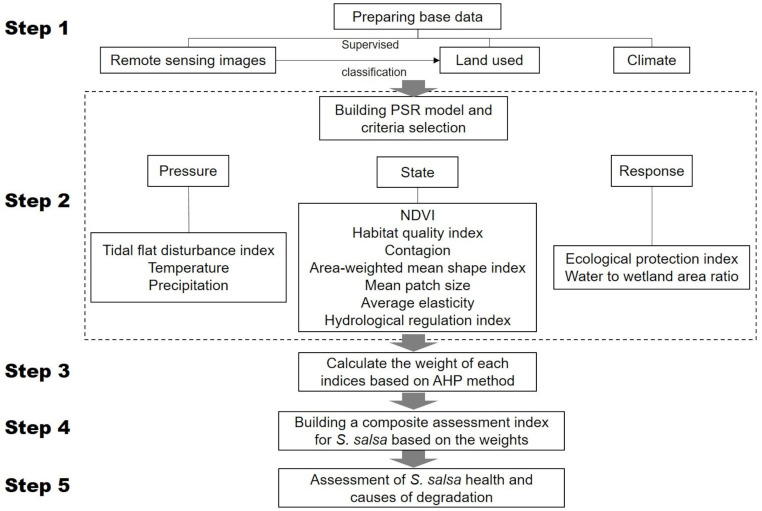
Technical process framework for the construction of the PSR model.

At present, many research methods have been used to assess wetland degradation, each of which has its advantages and scope of application. The integrated evaluation method is widely used in the evaluation of wetland ecosystem health because of its equivalence, ease of comparison and simplicity [43,44]. In order to scientifically and effectively evaluate the health status of *S. salsa* from 2016 to 2019, a PSR model-based health assessment index was constructed using the weights calculated by AHP, and many studies have been conducted to prove the universality and effectiveness of this method [12,16,17,19,20]. Firstly, the pressure index, state index and response index of the PSR model were calculated, after which the weighting of the above indices was used to obtain a composite assessment index of the wetland *S. salsa* community. The lower the index value, the more serious the degradation of the *S. salsa* community. Figure 2 shows the framework of the process of constructing a PSR model to assess the health status of *S. salsa*.

The formula of the comprehensive evaluation method is as follows:

(1) $$P_{i}=\sum_{j=1}^{n}W_{j}*X_{j}$$

where *P_i_*: the comprehensive evaluation value of the *i*th evaluation area; *W_j_*: the weight of the *j*th index; and *Xj*: the standardized value of the *i*th indicator.

## 3. Results

### 3.1. Landscape Pattern Change of the Liao River Estuary National Nature Reserve

**Figure 3:**
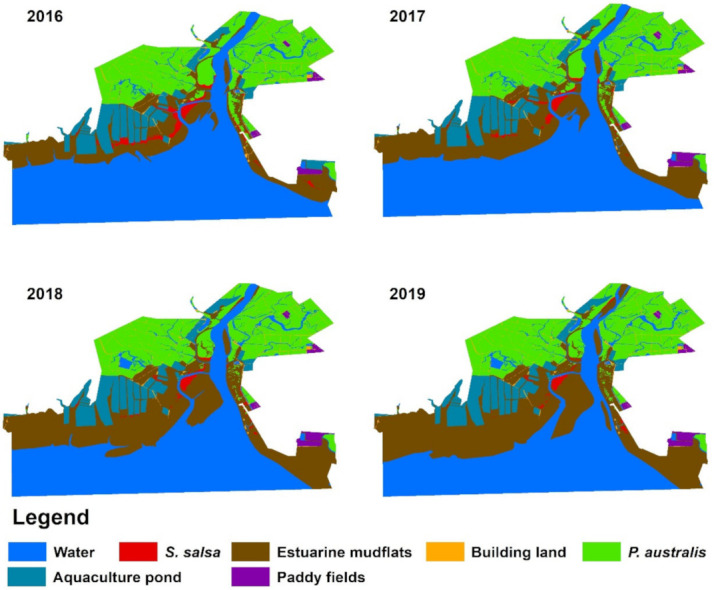
Changes in landscape patterns in the study area from 2016 to 2019.

The spatial and temporal changes in the landscape pattern of the Liao River Estuary National Nature Reserve from 2016 to 2019 are shown in Figure 3 and Table 5. *P. australis* were the most common plant in the reserve and were mainly distributed in the north of the study area. Small areas with both *P. australis* and *S. salsa* community were distributed on the east and west sides of the Shuangtaizi River. *S. salsa* was mainly distributed on the beaches on the east and west sides of the Shuangtaizi River. The results indicate that the *S. salsa* community shrank significantly from 8.027 km^2^ to 3.115 km^2^ from 2016 to 2019, and the loss rate reached 1.228 km^2^·year^−1^. It should be noted that the shrinkage of *S. salsa* on the west side of the Shuangtaizi River was the most significant, and some contiguous *S. salsa* communities gradually became fragmented and finally disappeared. From 2016 to 2019, the area covered by reeds decreased from 123.089 km^2^ to 117.079 km^2^, with an average annual reduction of 1.5025 km^2^. The beach area in the study area has increased significantly, from 87.521 km^2^ in 2016 to 167.493 km^2^ in 2019, with an average annual growth of 19.993 km^2^. The open water area decreased from 271.737 km^2^ to 201.917 km^2^. The area covered by artificial surfaces, aquaculture ponds and paddy fields did not change. The reduction in the area of *P. australis* and *S. salsa* is associated with the expansion of mudflats, which may be related to the accumulation of sediment carried by rivers and the reduction of water bodies on land. Spatially, it can be seen that the large reduction of water bodies changed to estuarine beaches, which may be related to the reduction of the *S. salsa* community.

### 3.2. Assessing S. salsa Community Health from Pressure, State and Response Indicators

**Figure 4:**
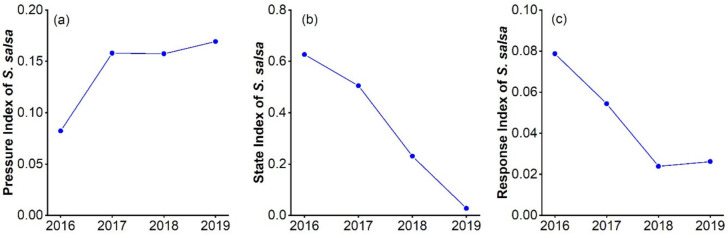
(**a**) pressure indicators, (**b**) state indicators and (**c**) response indicators for the PSR model from 2016 to 2019.

As can be seen in Table 6, the mudflat disturbance index increased each year from 2016 to 2019, with the rate of increase slowing from 2018 to 2019. In contrast, the temperature and precipitation indices fluctuate slightly but do not change much. This suggests that precipitation and temperature may have little effect on *S. salsa* during the four-year period. Figure 4a shows that the pressure index of the *S. salsa* community exhibits an upward pattern, and the increasing trend mainly had the contribution of the expansion of the mudflat. In summary, it can be shown that the exposed mudflats caused by the retreating water bodies put pressure on the health of the *S. salsa* community. The expansion of the mudflat area may reduce the water content in the mudflats away from water bodies, and the salt in the deposited sediment may be subjected to capillary forces that cause surface aggregation, resulting in a rise in salinity in the root zone of *S. salsa* and affecting seed germination and growth. Therefore, the increase of the mudflat area may affect the growth of *S. salsa* due to the change of soil salinity and moisture.

It can be seen in Table 5 that the NDVI value of *S. salsa* increased by 0.021 from 2016 to 2017 and decreased significantly from 2017 to 2019, with an average annual decrease of 0.11. The results show that the year-on-year declines in the habitat quality index, mean resilience and hydro regulation index indicate a progressive deterioration of the *S. salsa* community. The results showed that the habitat quality index decreased year by year, indicating that the survival environment of the *S. salsa* community gradually became poor. The deterioration of the habitat may be due to various reasons, such as the increase of the mudflat area, the increase of a particular species and human activities. The expansion of the increased mudflat area may affect the soil water and salt changes and coercevegetation growth; the proliferation of some herbivores such as crabs may also affect the health of *S. salsa*. The development of tourism may be reflected in the increase of artificial facilities and garbage, mainly in the area east of the inlet. In particular, abandoned aquaculture ponds and associated hydraulic facilities can form separate hydrological response units and affect the water content and salinity of the exposed mudflats in their vicinity, thus affecting the growth of *S. salsa*. The three indices, namely, the contagion, the weighted average shape factor and the average patch area, could well express the structure of a landscape. In 2016 to 2018, the contagion decreased year by year, with a total decrease of 5.589. However, the contagion suddenly increased by 2.963 in 2018 to 2019 because some patches in the landscape disappeared in 2019. The contagion indicates that the *S. salsa* community is well connected, but there is a decreasing trend of extension. The year-on-year decrease in the Area-weighted mean shape index illustrates the diminished complexity of the spatial pattern of *S. salsa*.

According to the above seven indicators and the weight calculated by the AHP model, the state index of the *S. salsa* community in 2016 to 2019 was obtained, see Figure 4b. The state index of the *S. salsa* community showed a downward trend. Following a series of external pressures, wetland ecosystems respond to stresses and changes in the condition due to changes in the landscape pattern and environmental condition of the wetland. Two indices, the ecological protection index and the Water to wetland area ratio, indicate that the *S. salsa* community responded to the pressures with a year-on-year decrease of 0.448 and 2.234 cumulatively, respectively. The decrease in the ecological protection index indicates that anthropogenic measures of ecological restoration have been ineffective.

According to the above two indicators and the weight calculated by the AHP model, the response index of the *S. salsa* community from 2016 to 2019 was obtained (see Figure 4c). The response index of the *S. salsa* community showed a downward trend. The response index is a consistently positive indicator, with decreases indicating gradual destruction.

### 3.3. Composite Health Index of the S. salsa Community

**Figure 5:**
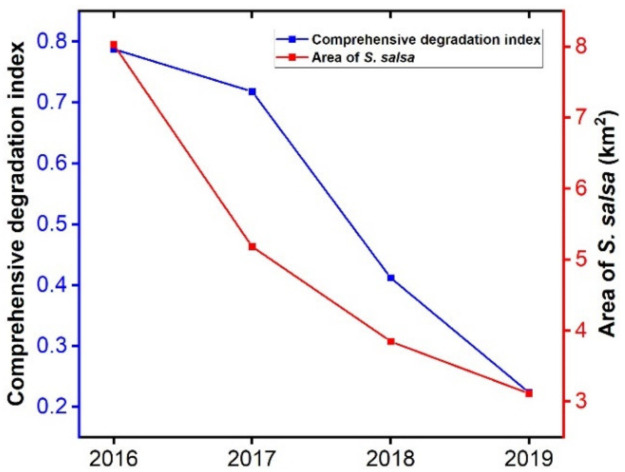
Comprehensive health index and area of *S. salsa* in wetlands from 2016 to 2019.

The combined health index of the *S. salsa* community from 2016 to 2019 is shown in Figure 5. The health index decreased from 0.788 to 0.233 over the four years, an average increase of 2.195 per year. The area of the *S. salsa* community decreased from 8.027 km^2^ to 3.115 km^2^, and the rate of loss of *S. salsa* was 1.228 km^2^ per year^−1^. The correlation between the health index of the *S. salsa* community and the area of *S. salsa* was 0.890. The composite health index of the S. salsa community constructed in this study shows a good correlation with the area of the S. salsa community, which can effectively indicate the health status of *S. salsa*.

In summary, the results of the constructed stress state response model assessment show that the *S. salsa* community is still in degradation, although aquaculture ponds pressure on the nature reserve is no longer applied and conservation measures have been taken. This indicates that the previous damage has severely damaged the local ecosystem and that the natural recovery capacity of the wetland ecosystem cannot restore the *S. salsa* community to its original state.

## 4. Discussion

In order to bring back the “red beach” landscape and restore the environment suitable for the survival of *S. salsa* in the Liao River Estuary National Nature Reserve, Panjin City carried out a wetland restoration project of “returning the land to the beach” in 2015. However, according to the results of this study, the area of *S. salsa* communities in the Liao River estuary continued to decrease from 2016 to 2019, from 8.027 km^2^ to 3.115 km^2^, with a loss rate of 1.228 km^2^·year^−1^. Part of the original *S. salsa* community was converted to bare mudflats.

Since 1992, aquaculture ponds have been constructed by local residents within the reserve to increase economic income. Until 2015, the expansion of aquaculture was an important factor in the degradation of the *S. salsa* community. Although no new aquaculture ponds were constructed after 2015, abandoned aquaculture ponds also remained in place. However, it is undeniable that the existing abandoned aquaculture ponds continue to have a negative impact on the survival of the *S. salsa* community. This is because the *S. salsa* community on the eastern side of the inlet near the aquaculture ponds is degrading faster than that on the western side. Another theory is that herbivorous crabs can adversely affect the growth of *S. salsa*, but the source of crabs may be abandoned breeding ponds [45].

*S. salsa* grows on both sides of the coastal tidal ditch or in the low-lying areas affected by the tide. Generally speaking, the main environmental factors affecting the growth of *S. salsa* are soil salinity and water content [46]. The self-regulation of *S. salsa* allows it to generate a range of salinities, but, if the salinity is too low, it will not turn red and lose its tourist value, while, if the salinity is too high, it will directly lead to the death of *S. salsa* [47]. Some previous studies have suggested that human activities, including some economic activities such as land use changes, have led to the ecological degradation of coastal wetlands in China [6,48]. However, Li et al. [49] used hydrodynamic modeling and statistical methods to find that limited tidal amplitude may be responsible for wetland degradation. The closure of the gates resulted in a low-flow water flow environment, where the evaporation of water and scarce freshwater recharge led to the deposition of soil salts in the upper layers of the mudflats. This may have contributed to the degradation of *S. salsa* and coincides with the decrease in the hydrological regulation index in this study. Changes in tidal amplitude can also lead to changes in the shallow water table, which further leads to changes in the water content and salinity of the upper soil layer, thus affecting the health of the wetland vegetation [50]. In summary, therefore, the degradation of *S. salsa* communities may be caused by anthropogenic activities such as abandoned aquaculture ponds, sluice management systems and changes in water salinity from tidal amplitudes and exposed mudflats.

Environmental changes such as soil salinity due to sea level rise caused by climate change can also adversely affect saline vegetation in coastal wetlands [51]. However, the shoreline of the mudflats in the Liao River estuary pushed toward the ocean from 2016 to 2019 is mainly influenced by the sediment carried by rivers [52]. The change of beach area leads to the change of beach salinity and water content, which in turn affects the growth of *S. salsa*. Therefore, sea level rise is not the main factor for the degradation of *S. salsa* in the Liao River estuary, but the change of soil salinity due to climate change, which affects the vegetation, cannot be excluded [53]. Another concern is the impact of tourism on the wetlands of the Liao River estuary, such as the construction of viewing corridors and garbage [54].

The cultural and ecological services of the S. salsa community are mainly reflected in its tourism value, where its high landscape aesthetics bring ornamental value while creating a healthy mood among tourists and generating income for the government [55]. Its ecosystem services are mainly in sand fixation and providing a good living environment for other organisms, such as birds and crabs. On balance, the over-exploitation of its cultural and ecological services can compromise its ecosystem services, so wetland managers need to be aware of this as a trade-off [56].

The present study constructed an integrated health index to evaluate the health of *S. salsa* communities based on the PSR model, which is similar to the study by Wang et al. [17] in that both can assess the health of a single vegetation cover. However, the limitation of this study is the lack of measured soil water content and salinity data, especially in areas where *S. salsa* community degradation occurs. However, as the mechanism of water and salt transport in the soil is clear, especially in areas with shallow groundwater depths, and salt in the surface soil is mainly influenced by capillary forces, it is generally feasible to construct a PSR model to assess the health of *S. salsa* communities and to analyze the causes of their degradation.

## 5. Conclusions

In this work, we tracked the dynamic changes of *S. salsa* in the Liao River Estuary National Nature Reserve from 2016 to 2019 by classifying and monitoring Sentinel-2 historical images and assessed the health status of *S. salsa* using a PSR model. The PSR model can effectively assess the health status of single vegetation in the estuary wetlands. The results showed that, despite the ecological restoration program in 2015, the *S. salsa* community was still in a state of decline year to year. From 2016 to 2019, the *S. salsa* community in the study area decreased from 8.027 km^2^ to 3.115 km^2^ and was mainly converted to bare mudflats. The causes of *S. salsa* community degradation include anthropogenic impacts from abandoned aquaculture ponds and sluice control systems but also natural impacts from changes in the tidal amplitude and soil properties of the mudflats. Anthropogenic interventions including the establishment of experimental sites and bio-grids are necessary to restore the *S. salsa* community to its original state. We recommend that the impact of existing human activities (e.g., aquaculture ponds) be taken into account when implementing ecological restoration projects and that the cultural and ecological services of the *S. salsa* community be weighed against the ecosystem services. The results of this study can provide a scientific reference for the management of vegetation and ecological restoration projects in the estuary wetlands.

## Figures and Tables

**Table 1:** Information about the selected satellite image.

Year	Images Acquisition Data	Cloud Coverage (%)
2016	22 August 2016	<10%
2017	25 August 2017	<10%
2018	25 August 2018	<10%
2019	25 August 2019	<10%

**Table 2:** Overall accuracy, Kappa coefficients and User accuracy of image classification.

Year	Overall Accuracy	Kappa Accuracy	User Accuracy
2016	0.999	0.999	0.917
2017	0.999	0.999	0.933
2018	0.998	0.998	0.967
2019	0.998	0.998	0.917

**Table 3:** Parameters and sources of the PSR model evaluation system.

Criteria	Indicator	Data Sources
Pressure	Tidal flat disturbance index	The area of river changing into tidal flat
	Temperature change	China Meteorological Data Service Center
	Precipitation	China Meteorological Data Service Center
State	NDVI	$\left(NIR-RED\right)/\left(NIR+\mathrm{RED}\right)$
	Habitat quality index	$A_{w}\times \left(0.18L+0.23G+0.40B+0.08D+0.01E+0.1F\right)/S$
	Contagion	Fragstats4.2
	Area-weighted mean shape index	Fragstats4.2
	Mean patch size	Fragstats4.2
	Average elasticity	$\sum \left(S_{i}P_{i}\right)/S$
	Hydrological regulation index	$\left(0.7W+0.3T\right)/S$
Response	Ecological protection index	$0.50WL+0.50\left(100-KL\right)$
	Water to wetland area ratio	$A_{n}\left(0.6W+0.4T\right)/S$

Note: *NIR*: Near infrared band; *RED*: Red band; *A_w_*: The normalized coefficient of habitat quality index of wetland ecosystem type nature reserve is 511.264; *L*: Woodland area; *G*: Grassland area; *D*: Cultivated land area; E: Artificial urban construction area; *F*: Unused land area; *B*: Water wetland area = 0.6 × water surface area + 0.4 × river beach; *S*: Total area of the study area; *S_i_*: Area of type *i* features; *P_i_*: The elastic distribution value of type i feature; *W*: Water surface area; *T*: Beach area; *WL*: Suitable index of wetland area in water area = A × ((water surface + River Beach)/core area) A—normalization coefficient of suitable area index, with a reference value of 100; *KL*: Development interference index = A_n_ × (artificial surface/core area); A_n_: The normalized coefficient of water wetland area ratio and the reference value of water conservation ecological function area is 102.722.

**Table 4:** The correlation matrix and weights of the indicators using the AHP method.

Higher-Level Indicator	Lower-Level Indicator	Judgement Matrix							Priority	Weight
*S. salsa* health	Pressure	1	1/3	3					0.258	
	State	3	1	5					0.637	
	Response	1/3	1/5	1					0.105	
Pressure	Tidal flat disturbance index	1	3/4	3/4					0.4	0.103
	Temperature change	4/3	1	1					0.3	0.077
	Precipitation	4/3	1	1					0.3	0.077
State	NDVI	1	1	3	3	3	1	1	0.2	0.127
	Habitat quality index	1	1	3	3	3	1	1	0.2	0.127
	Contagion	1/3	1/3	1	2	2	1/3	1/3	0.081	0.052
	Area-weighted mean shape index	1/3	1/3	1/2	1	1	1/3	1/3	0.06	0.038
	Mean patch size	1/3	1/3	1/2	1	1	1/3	1/3	0.06	0.038
	Average elasticity	1	1	3	3	3	1	1	0.2	0.127
	Hydrological regulation index	1	1	3	3	3	1	1	0.2	0.127
Response	Ecological protection index	1	3						0.75	0.079
	Water to wetland area ratio	1/3	1						0.25	0.027

**Table 5:** Area of each type (km^2^).

Type	2016	2017	2018	2019
Water	271.737	255.755	221.423	201.917
Estuarine mudflats	95.548	112.598	150.756	170.593
Building land	7.279	7.172	7.209	7.209
*P. australis*	123.089	121.804	117.411	117.079
Aquaculture ponds	44.988	43.078	43.669	43.669
*S. salsa*	8.027	5.185	3.851	3.115
Paddy field	3.675	5.907	5.907	5.907

**Table 6:** Information on each indicator of the PSR model from 2016 to 2019.

Criteria	Indicator	2016	2017	2018	2019
Pressure	Tidal flat disturbance index	2.543	17.344	35.712	45.301
	Temperature change	11.41	12.07	11.26	11.953
	Precipitation	76.28	72.15	77.98	64.1
State	NDVI	0.335	0.356	0.283	0.117
	Habitat quality index	48.539	47.20821	44.823	43.617
	Contagion	59.372	58.328	53.783	56.746
	Area-weighted mean shape index	14,426.837	13,162.932	10,943.731	7306.996
	Mean patch size	25.573	25.328	25.324	25.255
	Mean resilience	0.766	0.764	0.759	0.755
	Hydroregulation index	0.369	0.364	0.354	0.347
Response	Ecological protection index	50.389	50.403	50.472	50.863
	Water to wetland area ratio	37.864	37.343	36.340	35.631
